# Physical Therapy Combined with Transcranial Magnetic Stimulation Therapy: Treatment Practice Considering the Effect of Reducing Upper Limb Spasticity on Gait

**DOI:** 10.1298/ptr.R0025

**Published:** 2023-05-30

**Authors:** Yasuhide NAKAYAMA, Masahiro ABO

**Affiliations:** ^1^Department of Rehabilitation Medicine, The Jikei University School of Medicine, Japan

**Keywords:** Repetitive transcranial magnetic stimulation, Hemiplegia, Physical therapy, Rehabilitation

## Abstract

We perform physical therapy combined with repetitive transcranial magnetic stimulation (rTMS) in stroke patients with hemiplegia in the maintenance phase with the intent of improving the support of paralyzed leg. In gait evaluation in patients with hemiplegia, it is important to assess elements related to coordination carefully. rTMS therapy is effective in alleviating the tension of upper limbs. As rTMS helps upper-limb swing to become evident during gait, it makes trunk rotation necessary for left–right coordination appear more easily. As a result, rTMS has potential for improved upper-limb swing or trunk rotation. Post-rTMS therapy may prepare for the environment suitable for hip extending the stance phase of the paralyzed side. In physical therapy, it is advisable to practice standing up, maintaining standing posture or walking by making good use of these effects. We conduct practices in combination with the following: standing up focusing on load evenly distributed on both sides, standing on slant-board training, which enables forward shift of center of mass, walking by fixating upper limbs to the back of the body with the intent of extending the stance phase of the paralyzed side, and increasing trunk rotation. It is also necessary to discuss the combination with injection with botulinum toxin, which suppresses spasticity of ankle plantar flexors with the physician. Gait is associated with a variety of factors and has significant intrapatient and interpatient variations. In this regard, physiotherapists are required to develop a treatment program based on a quantitative evaluation, especially, in patients with hemiplegia.

**W**e have so far performed physical therapy combined with repetitive transcranial magnetic stimulation (rTMS) in stroke patients with hemiplegia in the maintenance phase actively with the intent of improving the support of paralyzed leg naturally.

Gait is described as the efficient spatial shift in the center of gravity (COG)^1)^. COG can be translated into the center of mass (COM) of a body, and it is discriminated from the center of pressure produced by the ground reaction force at times. Human can walk forward by putting force on the ground by plantar aspect, controlling joints ideally, preventing the body from swaying right to left or up and down, and reaction force applied to the ground. Gait can be divided into two phases: “stance phase” and “swing phase” in terms of kinetics, which is dynamic characteristics of gait. The stance phase represents about 60% of the gait cycle, while the swing phase occupies around 40% of it. The double stance phase represents about 20%. Each gait cycle begins at initial contact with a stance phase, followed by loading response, mid stance, and terminal stance, and proceeds through a swing phase: pre-swing, initial swing, mid swing, and terminal swing. Thus, it is common to observe and analyze the gait based on this cycle step by step^2)^. The flow of repeating the stance phase following the swing phase is a major characteristic of the standard gait. As this rhythmical gait cycle is disrupted in patients with hemiplegia, evaluating the coordination carefully is the first step toward the treatment of hemiplegia.

## Getting Faster Maximum Gait Speed Is Not a Treatment Effect

Kinematic motion analysis is a key to approach for gait provided by physiotherapists. On the other hand, spatiotemporal analysis is also clinically useful and is widely used for the diagnosis and judgement of the treatment effect. The most well-known evaluation method is the 10-meter walk test used to measure walking speed over 10 m^3)^. For the relationship between the gait speed and the gait function in hemiplegic patients, <0.4 m/s predicts household walking, 0.4 to 0.8 m/s predicts limited community walking, and >0.8 m/s predicts unlimited community walking^4)^. A change of getting faster gait speed by using differences between pretreatment and posttreatment is often used as a measure of improvement. However, in terms of kinematic motion analysis, this outcome measure should be interpreted very carefully. This is why 10-meter walk is measured with a maximum effort in most cases. For gait in healthy people, when the gait speed becomes faster, both the stance phase and the double stance phase decrease and the swing phase increases. If it were hemiplegic patients, it might lead to the further decrease in the stance phase of paralyzed leg than in the healthy individual. Alternating the stance phase and the swing phase smoothly is coordination. Rebuilding smooth performance is a treatment goal in physical therapy. However, the supporting phase of paralyzed leg may be increasingly decreased because patients have a conscious desire to increase gait speed. As the reduced supporting time of the paralyzed leg causes loss of activities without fail, leading to the atrophy of paralyzed muscles, it must be avoided. Also, it is necessary to keep in mind to develop a program to enable COM shift and joint control smoothly, not only to extend the supporting period of the paralyzed leg. We herein report changes in the gait of hemiplegic patients using rTMS, which we actively perform in our hospital as well as physical therapy.

## Evaluation in Performing Physical Therapy in Combination with rTMS

The gait of hemiplegic patients is characterized by increased stance phase and decreased swing phase when compared with healthy individuals^5^^–^^7)^, which causes asymmetric gait patterns. Also, it is known to increase the double stance phase gradually^8^^–^^10)^. Hemiplegic patients have difficulty with rotation toward the opposite direction of pelvis and chest^11)^. As rTMS therapy is effective in alleviating the tension of upper limbs^12)^, it helps upper-limb swing to become evident during gait and makes trunk rotation necessary for left–right coordination appear more easily^13)^. As a result, rTMS has potential for improved upper-limb swing or trunk rotation. Post-rTMS therapy may prepare for the environment suitable for extending the stance phase of the paralyzed side. In hemiplegic patients, it is difficult to support the load due to the impaired function of ankle plantar flexors or toe flexor muscles in the COM shift to forefoot in the terminal stance of the paralyzed side^14)^. In the physical therapy, it is necessary to develop a kinetic therapy that enables the movement of ankle plantar flexors during the prone hip extension and the slightly flexed position of the knee and the shift of the COM anteriorly by focusing on the shift of the body weight to forefoot in the stance phase of the paralyzed side.

Most of the patients give a priority to the stability and touch the ground by the entire plantar aspect to avoid touching the ground by toe or in inversion position of the foot. In order to touch the ground by the entire plantar aspect, it is required to extend the stance duration of the lower limb on the non-paralyzed side and attempt touching the ground slowly as if choosing a touching place. For this purpose, it is also necessary to allow the patient heel to touch the ground using orthosis as needed. Patients with partial loss of sensation of plantar flexors tend to bend forward inevitably because they use visual compensation by looking at their foot. Hemiplegic patients rely on their vision to maintain standing balance when compared with healthy individuals^15)^. The use of a mirror is useful not only in entering sensory information but also in correcting their posture. It is recommendable to hear sensory impairment from them and provide feedback accordingly, and prepare the environment that allows patients to learn somatic positional relationship and how to apply force as much as possible by exploiting all their residual sensations. It is also important to establish the environment for feedback by setting gait width as narrow as possible.

Load applied to the forefoot on the paralyzed side may induce knee buckling because the load line shifts ahead of the axis of the knee. As patients always feel fear of knee buckling, they tend to prevent their weight from shifting forward as much as possible. The way of applying their body weight without fear is a strategy of locking knee joint in extension position. The patients can acquire the stability of their gait when the knee joint is locked in the extension position during the stance phase. The stronger the recoil is generated in locking, the more frequently genu recurvatum will be induced. The stance phase will inevitably occur without participation of knee extensors. As a result, it causes atrophy in quadriceps not in proportion to exercise volume. Once one remembers locking, it is difficult to correct. In case of severe paralysis, there are not a few patients who cannot support their weight without locking. It is essential to judge how much they can achieve by practicing standing up or walking from the early stage while attempting maximum sensory integration. It is necessary to prepare as much information that can be given to patients as possible as follows: providing visual feedback by making the utmost use of a mirror, giving appropriate support for the upper limb on the non-paralyzed side, and providing feedback using knee brace or surface electromyography in order not to transfer to knee hyperextension in the end range.

## Physical Therapy to Be Conducted as a Therapeutic Method Following rTMS

We provide a program that can exploit the effectiveness of rTMS therapy in addition to stretching following rTMS at our university. We describe herein our practical physical therapy.

### Standing-up practice focusing on left and right loads

Standing-up motion is a motion that shifts COM upward smoothly until the standing position is taken by putting the COM on the base of support consisting of buttock through plantar aspects of both feet. Patients with hemiplegia often stand up depending on the extremity on the non-paralyzed side dominantly^16^^,^^17)^. When paralysis becomes severe, it is extremely difficult to apply a load on the forefoot on the paralyzed side. Patients perform standing-up motion while applying their weight evenly distributed over both legs using visual feedback. Also, the decrease in load efficiency on the limb on the paralyzed side was assessed. A full-length mirror is always installed in front of patients in order to enter the information properly. A straight line is drawn on the mirror, and a seal is applied on the patient body as a mark. Patients consciously practice so that the mark on their body can move upward smoothly along with the line on the mirror. This practice helps patients reset load sensation between right and left legs, which they so far have felt. In this practice, patients can easily take in information on left–right coordination by placing a soft ball between their knees. Similarly, they can realize tension of both hands and compensatory body motion more easily by folding their arms. As the tension of upper limbs is likely to elevate during the practice, it is important to position a therapist on the paralyzed side or posteriorly so that he or she can relax as much as possible and his or her feeling of fear for applying a load on the paralyzed side can be removed. Giving a sense of assurance to patients, “Never fall” is a mandatory condition in conducting the training. The training with parallel bars can also give a sense of assurance to them. It is also important to take data on ground reaction force in standing up and provide correct information for patients.

### Standing on slant-board training (SST)

**Fig. 1.**
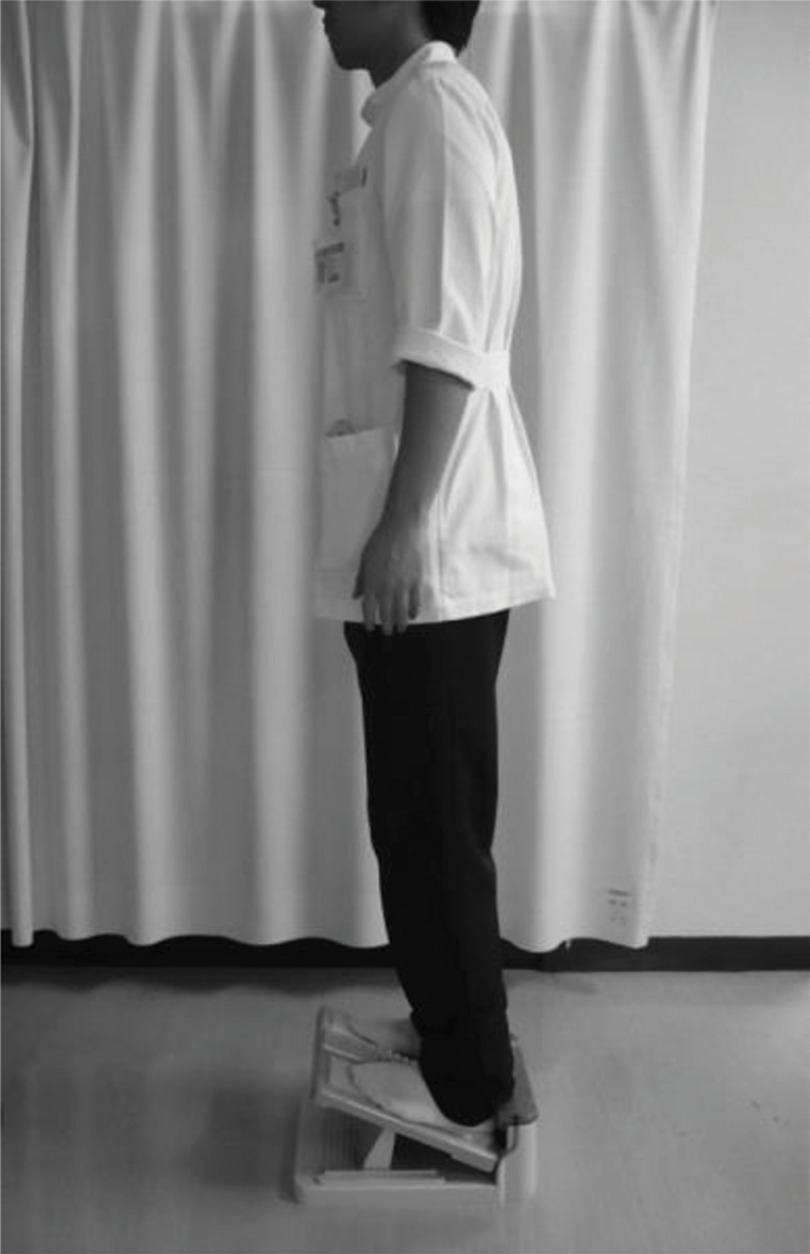
Standing on slant-board training The treatment is to maintain a standing posture on a slope with a toe up. In the subsequent standing position on the level ground, the weight center position deviated forward. This has been verified and reported in patients with hemiplegia and Parkinson’s disease.

It is required to change potential energy to kinetic energy efficiently to shift the COM anteriorly. Generally, the gait that can be explained by the inverted pendulum model has a high energy efficiency^18)^. For hemiplegic patients, as attention is required for their anxiety about applying a load on the paralyzed limb, the load application on the extremity on the non-paralyzed side dominantly, knee buckling or toe’s getting caught, the COM is often positioned at the rear of the non-paralyzed limb. The lower limb muscle weakness affects the reduced force of shifting the COM anteriorly toward the paralyzed side^19^^,^^20)^. We often employ the SST (Fig. 1) as one of the programs following rTMS in order to facilitate the shift of the COM anteriorly as physical therapy. The SST allows patients to shift the COM anteriorly easily by taking the standing position on the slant board. It is expected to exert an effect that hemiplegic patients whose COM is shifted backward can move more easily. A study reports that the SST is effective in improvement of walking ability not only for hemiplegic patients^21)^ but also for patients with Parkinson’s disease^22)^. We performed SST combined with rTMS in hemiplegic patients and reported that the COM in walking was shifted forward^23)^. The SST enables the COM to shift forward in patients who feel anxiety for applying a load to the forefoot on the paralyzed side due to sensory disorder in the lower limb on the paralyzed side.

### Walking practice by fixating upper limbs to the back of the body in shoulder extension and internal rotation position

A treadmill is a device used for walking practice at a constant speed. Hemiplegic patients develop asymmetric walking pattern due to the shortened support phase of the paralyzed side^24)^. Walking practice using a treadmill increases step length^25^^,^^26)^. A device with ground reaction force sensor can give a variety of information on walking related to ground contact to therapists or patients. A lot of patients have a fear for moving belt. As is the case with hemiplegic patients with sensory disorder or disturbance of sensation who feel fear about the moving belt, this also applies to elderly people with reduced postural control function. It is necessary to adjust the speed and take time to determine the appropriate speed.

We develop and provide the NEURO (NovEl Intervention Using Repetitive TMS and Intensive Occupational Therapy) program consisting of rTMS, physical therapy, and occupational therapy in patients who have a high tendency to bend due to upper limb spasticity. In the NEURO protocol during 2 weeks of hospitalization, each subject received daily 40-min low-frequency rTMS and 240-min intensive therapeutic exercise. The rTMS was directed at the primary motor cortex of the patient’s healthy hemisphere at 2400 pulses a day at a low frequency of 1 Hz. The stimulation intensity was set at 90% of the resting motor threshold for the first dorsal interosseous muscle of the non-paralyzed side. The therapeutic exercise was a combination of one-to-one training for 120 min and self-exercising for 120 min. In physical therapy, we have intervention by utilizing the effect of reducing spasticity follow rTMS with the intent of gait reconstruction.

**Fig. 2.**
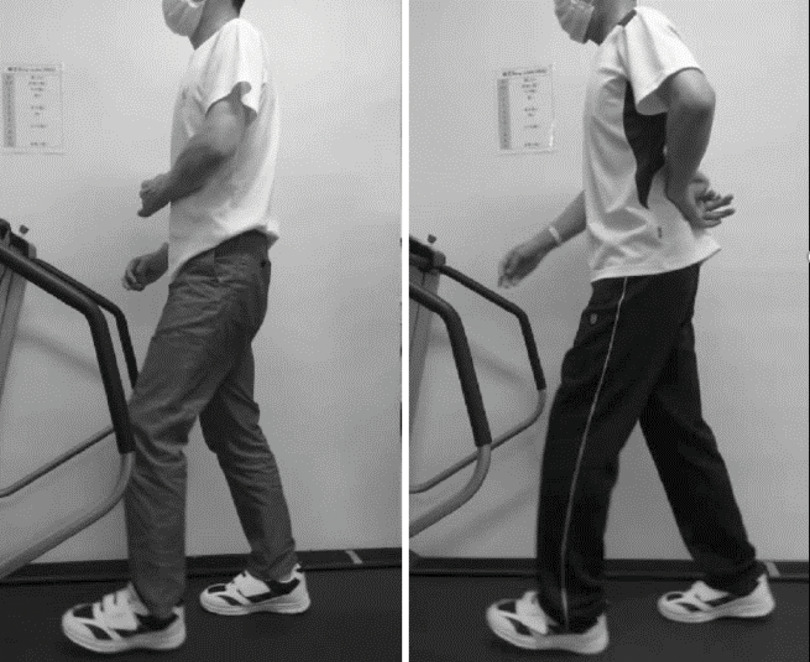
Normal gait practice (left) and gait practice with the upper limb immobilized on the back in shoulder extension internal rotation (right)

**Fig. 3.**
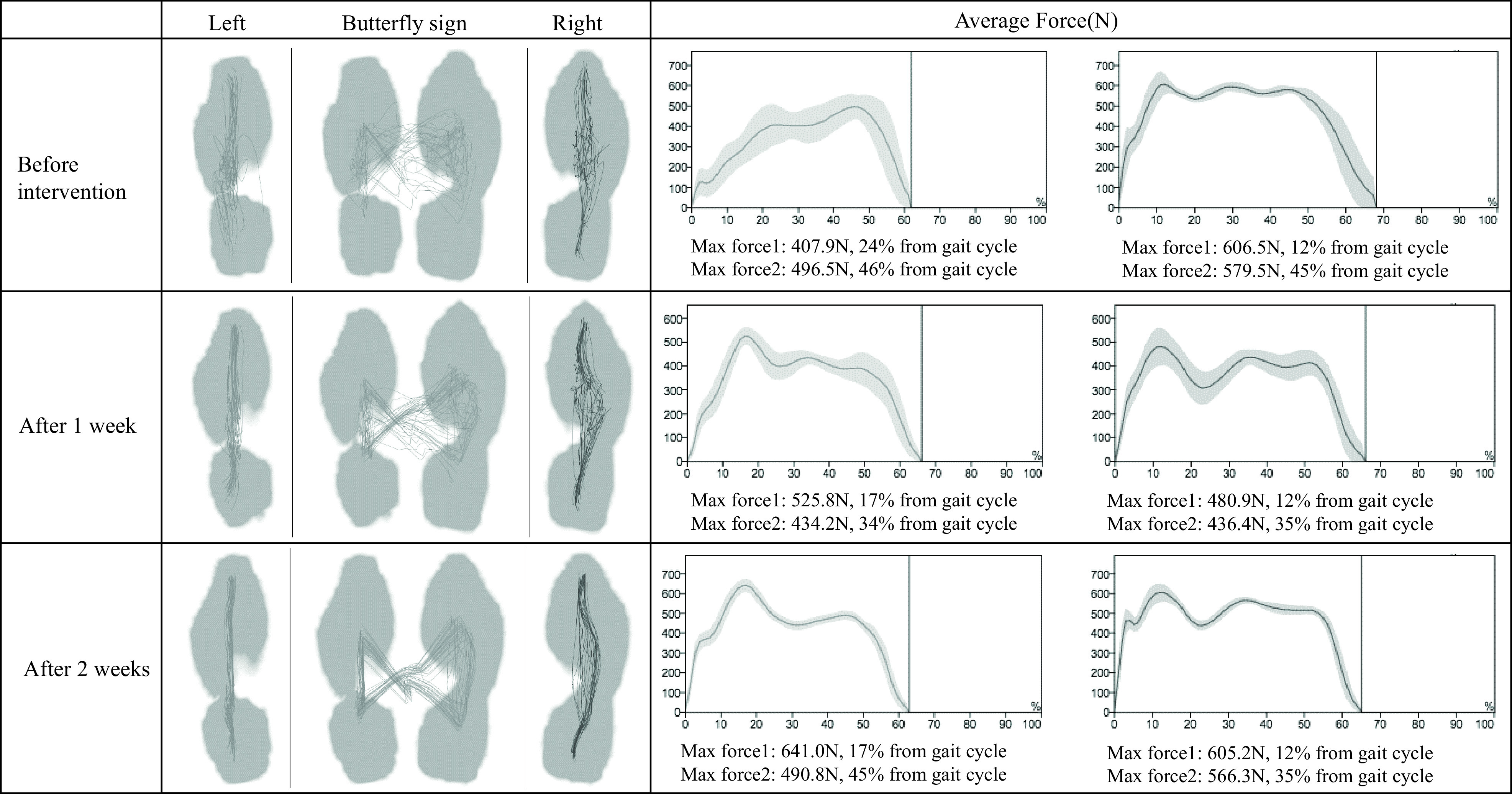
Butterfly sign and change in floor reaction force After 1 and 2 weeks, the data are from walking with the belt immobilization removed. The butterfly sign was gradually drawn and a bimodal effect could be seen in the floor reaction force.

It is demonstrated that walking practice by fixating upper limbs to the trunk using an arm sling (Fig. 2) enables load application to the paralyzed side more easily and that it extends the stance duration^27)^. However, a posture by positioning the upper limb anterior to the body with an arm sling shifts the COM backward. To deal with, we employ walking practice by fixating upper limbs to the back of the body with the belt. Fixating upper limbs to the back of the body inevitably shits the COM forward. The shoulder extension and internal rotation position are not frequently used in hemiplegic patients during walking. This training method is expected to improve the shift of COM forward, trunk rotation, and application of load to the paralyze side as well as load evenly distributed on both sides shown with a symbol of butterfly illustrated during normal walking (Fig. 3).

### Physical therapy combined with botulinum toxin

rTMS is known to reduce upper limb spasticity. While rTMS is reported to have a treatment effect on the lower limbs in terms of improvement in the gait speed^28)^, the improvement in gait patterns represents a challenge. We have so far investigated the impact of rTMS to the upper limbs on the lower limb function and also the effect of the combination with physical therapy. Some studies report that the spasticity of ankle plantar flexors has a huge impact on asymmetric walking pattern^29^^,^^30)^. It is shown that the use of botulinum toxin in rehabilitation therapy increases the walking ability of hemiplegic patients due to its effect to alleviate the spasticity of ankle plantar flexors significantly^31)^. In particular, the use of botulinum toxin in combination with rTMS has a high potential for realizing the load application in ankle dorsiflexion during the stance phase. It may be necessary to reconsider the indication or change in orthoses by controlling the movement of lower limb orthoses and understanding the impact of knee or hip joint during the terminal stance phase due to load application to the forefoot precisely. If sensory disorder is mild, it is possible to learn and apply a load in the ankle dorsiflexion position again.

## Improvement of Functional Impairment and Role of Physical Therapy Combined

In hemiplegic patients, recovery refers to the restoration of a function back to a normal state, whereas compensation refers to the substitution with a different state before its onset^32)^. Most patients with paralysis experience compensation. For kinetic therapy, it is required to choose a learning theory or trade-off concept on a case-by-case basis. Motor learning theory is a concept of neuropsychology aiming at automatization by giving the knowledge results (correct information) appropriately and by repeating practice. The motor learning theory is applied to a wide range of treatments. There are very few cases where physiotherapists assess whether patients can practice motor learning after being provided correct information. Motion analysis based on observations works out problems by breaking into more simple motion and comparing that with compound motion consisting of combination of individual simple motions. One must understand that the patients are always in the unstable state due to reduced separation motion or sensory disorder seen in paralysis. A program is needed to choose muscles whose activities should be suppressed or those whose activities should be elevated or for long-term planning of learning on the position of the COM.

## Conclusions

It is no exaggeration to say that independent gait depends on whether patients can live an independent life. In the aging society, rTMS stands as one of the therapeutic approaches to acquire smooth gait, which is widely recognized. Rationale is required for physical therapy performed in combination with rTMS. There are quite a few physical therapy modalities with unclear content despite advanced rehabilitation medicine. In rehabilitation medicine as aftertreatment program, one should keep in mind to understand which is physical therapy and to perform a certain treatment tailored to individual patient’s life by learning the patient lifestyle in the process of assessing meticulously and collecting information. It is important to provide therapy that physiotherapists can do after patients understand what paralysis is.

## Conflict of Interest

The author declares no conflicts of interest.
